# Predicting transient ischemic attack risk in patients with mild carotid stenosis using machine learning and CT radiomics

**DOI:** 10.3389/fneur.2023.1105616

**Published:** 2023-02-08

**Authors:** Hai Xia, Lei Yuan, Wei Zhao, Chenglei Zhang, Lingfeng Zhao, Jialin Hou, Yancheng Luan, Yuxin Bi, Yaoyu Feng

**Affiliations:** ^1^Department of Vascular Surgery, The First Affiliated Hospital of Kunming Medical University, Kunming, China; ^2^Department of Orthopedics, The First Affiliated Hospital of Kunming Medical University, Kunming, China; ^3^Imaging Intervention Center, The First Affiliated Hospital of Kunming Medical University, Kunming, China

**Keywords:** CT angiography, carotid artery, machine learning, prediction model, transient ischemic attack

## Abstract

**Objective:**

This study aims to establish a radiomics-based machine learning model that predicts the risk of transient ischemic attack in patients with mild carotid stenosis (30–50% North American Symptomatic Carotid Endarterectomy Trial) using extracted computed tomography radiomics features and clinical information.

**Methods:**

A total of 179 patients underwent carotid computed tomography angiography (CTA), and 219 carotid arteries with a plaque at the carotid bifurcation or proximal to the internal carotid artery were selected. The patients were divided into two groups; patients with symptoms of transient ischemic attack after CTA and patients without symptoms of transient ischemic attack after CTA. Then we performed random sampling methods stratified by the predictive outcome to obtain the training set (*N* = 165) and testing set (*N* = 66). 3D Slicer was employed to select the site of plaque on the computed tomography image as the volume of interest. An open-source package PyRadiomics in Python was used to extract radiomics features from the volume of interests. The random forest and logistic regression models were used to screen feature variables, and five classification algorithms were used, including random forest, eXtreme Gradient Boosting, logistic regression, support vector machine, and k-nearest neighbors. Data on radiomic feature information, clinical information, and the combination of these pieces of information were used to generate the model that predicts the risk of transient ischemic attack in patients with mild carotid artery stenosis (30–50% North American Symptomatic Carotid Endarterectomy Trial).

**Results:**

The random forest model that was built based on the radiomics and clinical feature information had the highest accuracy (area under curve = 0.879; 95% confidence interval, 0.787–0.979). The combined model outperformed the clinical model, whereas the combined model showed no significant difference from the radiomics model.

**Conclusion:**

The random forest model constructed with both radiomics and clinical information can accurately predict and improve discriminative power of computed tomography angiography in identifying ischemic symptoms in patients with carotid atherosclerosis. This model can aid in guiding the follow-up treatment of patients at high risk.

## Introduction

In the United States, 690,000 patients experience ischemic stroke yearly. Ischemic brain injury is caused by large-arterial atherosclerosis, cardioembolism, small-vessel disease, or cryptogenic cirrhosis. Carotid bifurcation plaques in atherosclerosis are prone to transient ischemic attack (TIA) and stroke. Among all patients with ischemic stroke, 173,000 (approximately 25%) suffer from atherosclerotic carotid artery disease (1). Endarterectomy and stenting are used to treat patients with 70–99% carotid stenosis (2–4), but moderate stenosis is more common and the main culprit of the plaques (5).

In addition, <50% of patients with stenosis are more difficult to treat and suffer from recurrent neurological disorders such as stroke and TIA (3). According to the 2017 European Society for Vascular Surgery (ESVS) clinical practice guidelines, patients with carotid stenosis who had cerebral infarction or TIA in the past 6 months were divided into symptomatic carotid stenosis and asymptomatic carotid stenosis groups. Best medical therapy (BMT) is recommended for patients with asymptomatic carotid stenosis with <60% stenosis and those with symptomatic carotid stenosis with <50% stenosis. In the past 5 years, the risk of stroke in 1,429 patients with 30–49% stenoses randomized to carotid endarterectomy (CEA) was 22.8%, compared with 25.5% on BMT. If symptoms persist, despite BMT, it is recommended to undergo carotid endarterectomy or carotid artery stenting (CEA/CAS) treatment (3).

Carotid computed tomography angiography (CTA) can measure carotid lumen stenosis and provide information about arterial wall calcification. While the stenosis degree provides crucial information about the disease, it does not determine the underlying plaque stability or inflammation degree and the probability of the patient experiencing a second event (6). Radiomics is a multi-step process that transforms medical images into high-dimensional structures to comprehensively analyze the regions of interest and correlate them with clinical, diagnostic, and prognostic information (7, 8). Standardized radiomics analysis comprises image acquisition, reconstruction, image preprocessing and processing, feature extraction, selection, and classification/regression modeling (6, 9, 10). Radiomics and machine learning are used to diagnose disease diagnosis and prognosis, limited to dermatology (11), oncology (12–14), and cardiac diseases (15). Radiological studies that evaluate carotid disease, especially in mildly stenotic carotid CTA imaging, are limited. Therefore, this study aims to identify patients' CTA region of interest and clinical information (6). Machine learning methods were used to establish a model that predicts the risk of TIA events with carotid artery stenosis by 30–50% and guides patients' follow-up treatment plans.

## Materials and methods

### Patients

**Figure 1:**
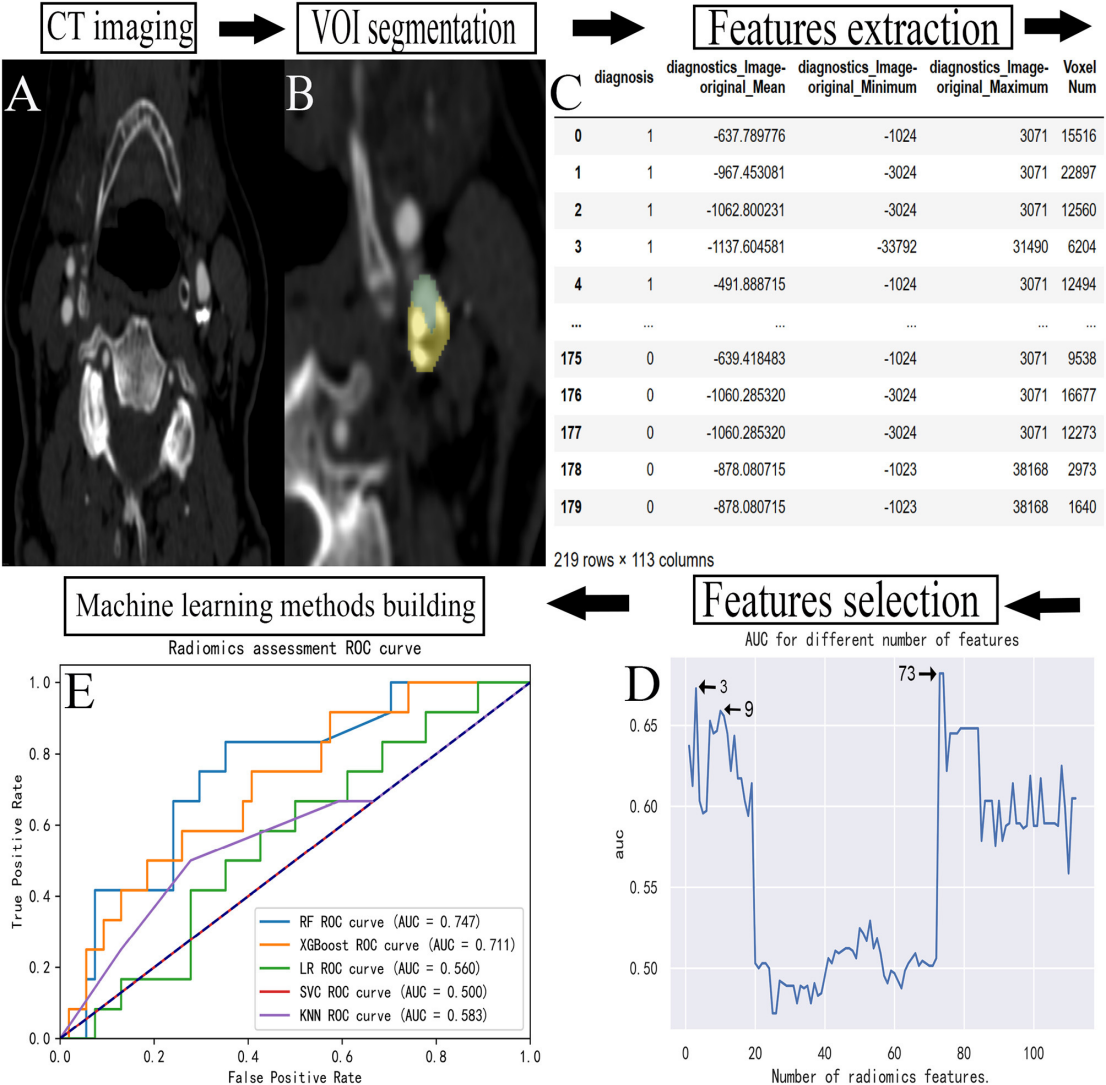
Flowchart for building a predictive model. **(A)** The carotid CTA image of a patient. **(B)** The manual VOI segmentation was performed on an image. **(C)** The radiomic features extracted from each VOI. **(D)** The feature data were selected. **(E)** The establishment of prediction models. CTA, computed tomography angiography; VOI, volume of interest; ROC, receiver operating characteristic; AUC, area under curve.

This study was approved by the ethics committee of The First Affiliated Hospital of Kunming Medical University (No. 2022-274). Written informed consent from all subjects (patients) was waived by the Ethics Committee of The First Affiliated Hospital of Kunming Medical University, because of the retrospective nature of the study. The flowchart of the current research protocol was presented in Figure 1. Nearly 615 patients with carotid artery stenosis were selected from October 2016 to March 2022. The inclusion criteria comprised patients undergoing carotid CTA and carotid artery stenosis of 30–50% with clinical laboratory tests (uric acid, triglycerides, low-density lipoprotein, homocysteine, and fibrinogen). The patients were excluded if they had (1) carotid artery dissection and aneurysm; (2) intracranial vascular disease (e.g., intracranial atherosclerosis with stenosis <50%, vasculitis, aneurysm); (3) posterior circulation stroke; (4) intracerebral hemorrhage; (5) other causes of hemorrhagic stroke (e.g., cardioembolic source and chest embolism); or (6) patients with other neurological diseases such as brain tumors and demyelinating diseases.

After the screening process, 179 patients were identified and 219 carotid arteries were included in this study. Follow-up continued until the outcome occurred or November 2022, whichever came first. Through follow-up, 34 patients (including 39 carotid arteries) developed TIA. There were no recurrences of stroke. The patients were divided into two groups: the symptomatic group: patients with symptoms of transient ischemic attack after CTA and the asymptomatic group: patients without symptoms of transient ischemic attack after CTA (Table 1).

**Table 1:** Stenosis degree and clinical features.

	**Non-TIA (*n* = 180)**	**TIA (*n* = 39)**	***P*-value**
Age (years)	65 ± 9.7	67.4 ± 10.4	0.181
Sex (%)			0.774
Female	53 (29.4)	13 (33.3)	
Male	127 (70.6)	26 (66.7)	
Diabetes (%)			0.277
No	146 (81.1)	28 (71.8)	
Yes	34 (18.9)	11 (28.2)	
Hypertension (%)			0.814
No	85 (47.2)	17 (43.6)	
Yes	95 (52.8)	22 (56.4)	
BMI (kg/m^*^2)	23.6 ± 2.6	23.3 ± 2.7	0.626
Smoking (%)			1.000
No	142 (78.9)	31 (79.5)	
Yes	38 (21.1)	8 (20.5)	
Antiplatelet and lipid lowering (%)			<0.001
No	105 (58.3)	7 (17.9)	
Yes	75 (41.7)	32 (82.1)	
UA (μmol/L)	362.0 ± 82.1	321.9 ± 75.8	0.006
TG (mmol/L)	1.7 ± 1.0	1.5 ± 1.4	0.405
LDL (mmol/L)	2.6 ± 0.8	2.1 ± 0.7	0.001
Fib (g/L)	3.2 ± 0.7	3.1 ± 0.8	0.635
HCY (μmol/L)	13.3 ± 5.4	15.0 ± 10.6	0.146
Carotid stenosis (%)	40.5 ± 6.4	39.9 ± 6.5	0.595

BMI, body mass index; UA, uric acid; TG, triglycerides; LDL, low-density lipoprotein; Fib, fibrinogen; HCY, homocysteine; TIA, transient ischemic attack. The results were compared using a t-test for continuous variables and a χ2 test for categorical variables. Continuous variables are shown with mean ± standard deviation (SD). Categorical variables were recorded as percentages.

Stenosis was measured according to the North American Symptomatic Carotid Endarterectomy Trial (NASCET) criteria (3). The symptoms were defined as patients who were considered symptomatic after a TIA or ischemic stroke in the ipsilateral cerebral hemisphere. TIA is defined as brief (24-h) episodes of neurological dysfunction, such as hemiplegia, sensory disturbances, dysarthria, speech disturbances, or monocular blindness.

### Image acquisition

Carotid computed tomography angiography imaging (Siemens SOMATOM Definition Flash DSCT) was used to scan the area from the aorta to the atlas. About 60–80 mL of non-ionic contrast agent Iopromide injection (360 mg/mL) and 50 mL of normal saline were injected from the cubital vein at a flow rate of 5 mL/s. Smart prep technology was used to observe the density of the targeted region in the aortic arch, and the scan was administered when the density reached 150 HU. The scanning parameters were tube voltage (120 kV), automatic tube current modulation technology, layer thickness (0.625 mm), layer spacing (0.625 mm), pitch (0.938:1), and rotation speed (0.5 r/s).

### Region of interest segmentation

**Figure 2:**
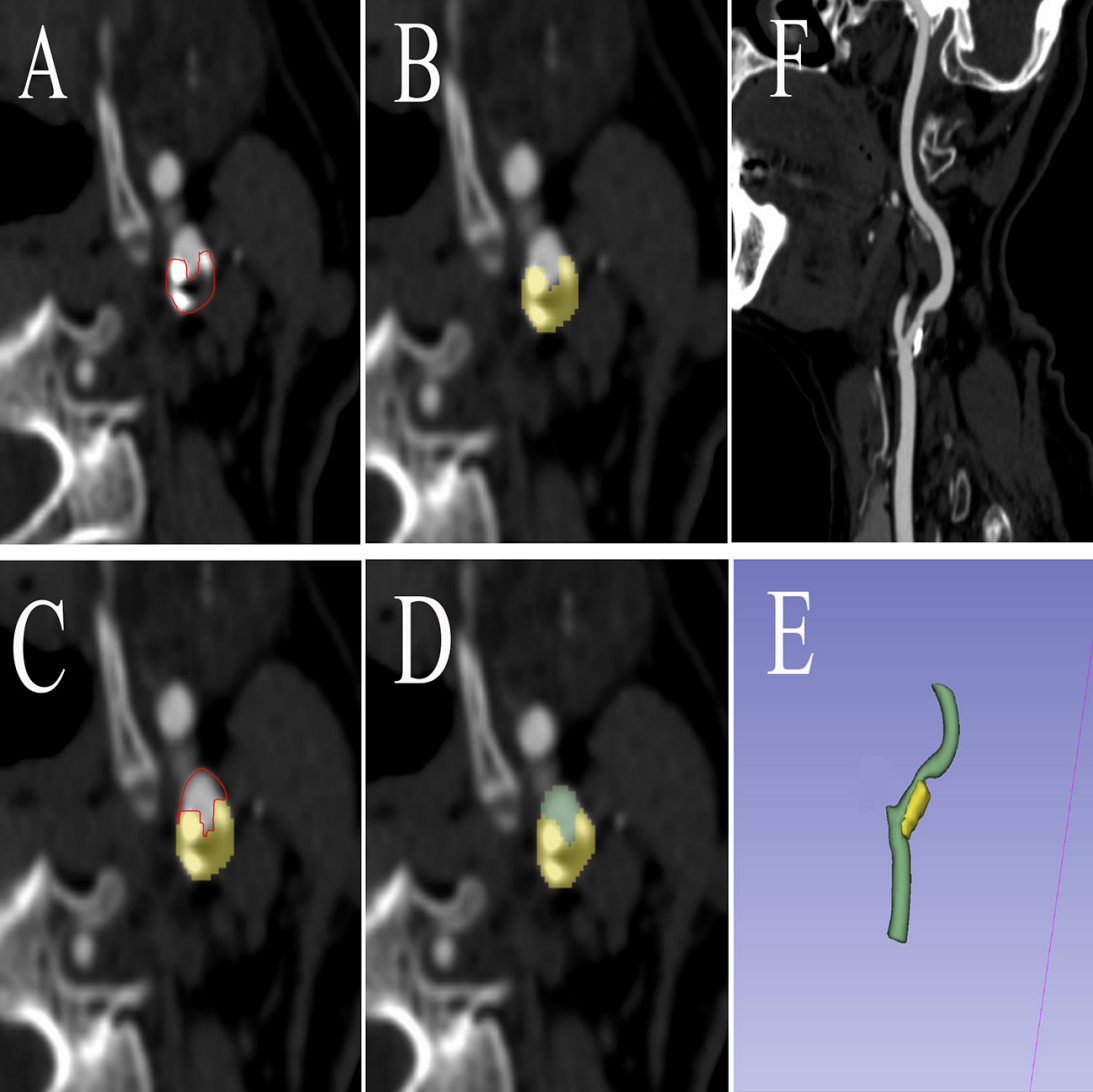
The procedure of radiomics segmentation. The VOI in each image was segmented with the green arterial lumen and yellow plaques, and a 3D model was synthesized. **(A)** The edges of atherosclerotic plaques were delineated. **(B)** Atherosclerotic plaques were segmented. **(C)** The edges of arteries were delineated. **(D)** Arteries were segmented. **(E)** A 3D model was built based on the segmentation of each layer image. **(F)** Carotid artery CTA corresponding image of the patient. VOI, volume of interest; CTA, computed tomography angiography.

Soft plaques have attenuation values of <50 HU. A mixed plaque is one that has attenuation values between 50 and 119 HU. Calcified plaques have attenuation values of >120 HU (16). During the image examination, window/level (window 900–1,200 HU, level 300–500 HU) to view calcified and non-calcified plaque. The calcified plaque was easily distinguished from non-calcified plaque by manually and visually manipulating the window/level. Vascular luminal contrast enhancement was easily separated visually from calcified plaque by the manual manipulation of window/level settings which allowed for optimal plaque component distinction (17). The manual volume of interest (VOI) segmentation was performed on all images using the 3D Slicer software package (version 4.11) (18). The carotid artery with arterial plaque was manually segmented into the targeted region. The proximal and distal carotid arteries were divided into normal vessels at least 2 cm away from the plaque. Figure 2 shows the sample patient images. The VOI of optimal size was accurately segmented to establish a precise relationship between the radiomics of carotid plaque and the transient ischemic attack incident.

### Radiomics feature extraction

An open-source package PyRadiomics (version 2.4, https://pyradiomics.readthedocs.io/) was used to extract the radiomic features. The radiomic features extracted from each volume were automatically calculated using Pyradiomics in Python. They were divided into eight groups: (a) first-order features, (b) shape features (3D), (c) shape features (2D), (d) grayscale co-occurrence matrix features, (e) grayscale size regions matrix function, (f) grayscale run-length matrix feature, (g) adjacent grayscale difference matrix feature, and (h) grayscale dependency matrix feature. A total of 129 radiomics features were extracted from each patient's VOI (19).

### Radiomics feature selection

The primary features that significantly impact the occurrence of TIA were identified by reducing the number of extracted features. About 129 radiomic features were converted into digital form through feature data. Seventeen features that could not be converted into digital form were excluded, and the remaining 112 features were included in the feature set. A feature selection algorithm based on the RF (20) method was used as the basic tool, incorporating the features into the importance order from high to low. Each time a feature was included, the optimal number of features was determined using the logistic regression model, and the classification area under curve (AUC) value was calculated. A total of 112 AUC values were calculated, of which the numbers of features corresponding to the top three AUC values were 73, 3, and 9, respectively. The prediction models were constructed with the features, respectively. The prediction performance of each model was optimal when 73 features were included, but it was not parsimonious. When only three features were included, the prediction performance of the subsequent prediction model was poor, thus, we chose to include the first nine features.

### Clinical features and combined features selection

The clinical features included four components, such as routine blood test data (triglyceride, low-density lipoprotein, homocysteine, uric acid, and fibrinogen (21–25), which were demonstrated to be closely related to carotid plaque formation according to previous studies), demographic data [age, gender and body mass index (BMI), high blood pressure, diabetes, and smoking (26–31)], stenosis of the carotid artery, and medication data [antiplatelet and lipid-lowering drugs (32)]. We performed the same process as radiomics feature selection. The final result was a model with better prediction performance when the first two features were included.

**Figure 3:**
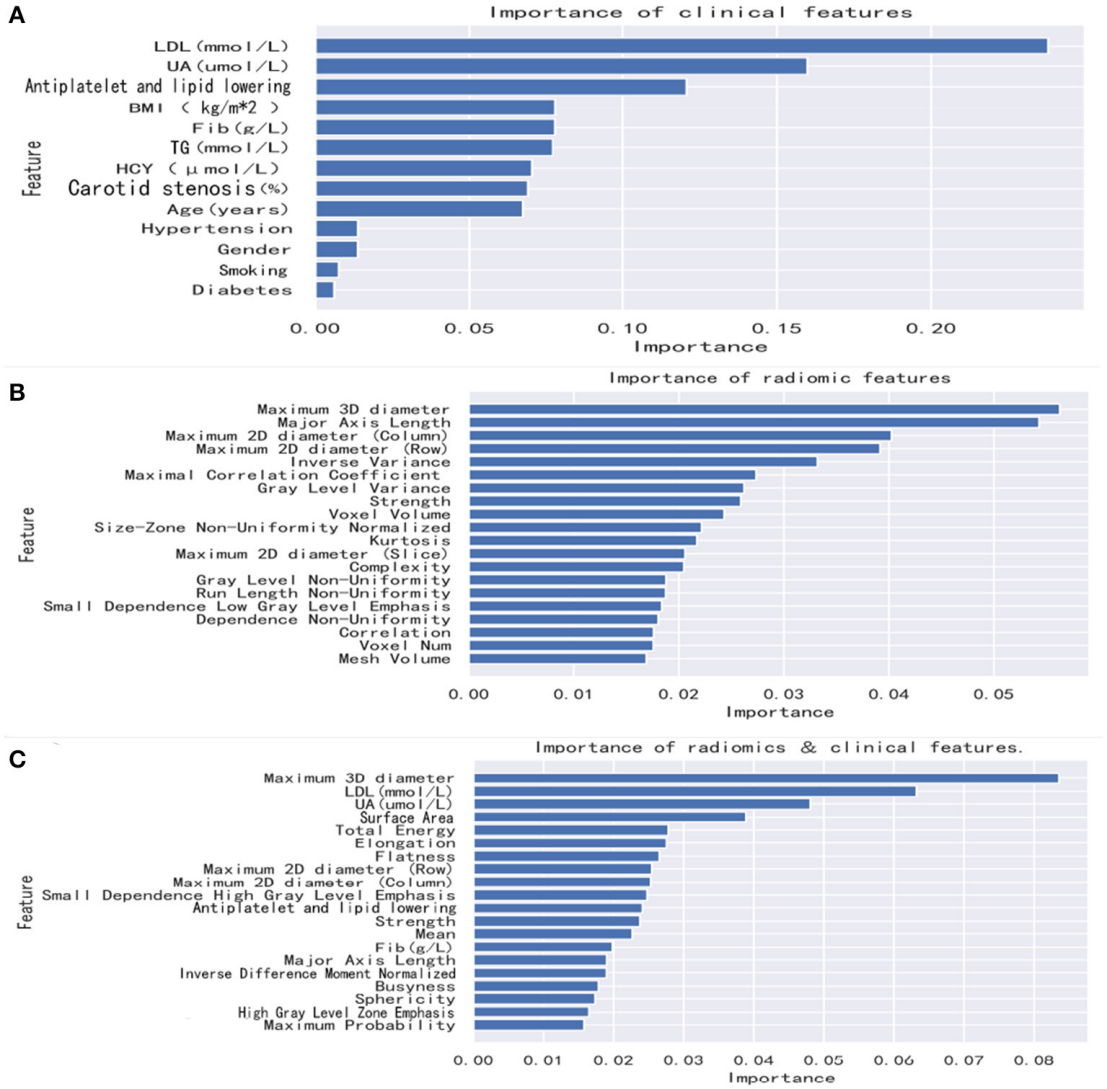
Feature importance in the random forest model. Radiomics features and combined features show only the top 20 features. Clinical features show all features. **(A)** The random forest method was used to rank clinical features from high to low importance. **(B)** The random forest method was used to rank radiomics features from high to low importance. **(C)** The random forest method was used to rank combined features from high to low importance. BMI, body mass index; UA, uric acid; TG, triglycerides; LDL, low-density lipoprotein; Fib, fibrinogen; HCY, homocysteine.

Similarly, as earlier, the radiomics and clinical feature data were merged into the same dataset, and feature screening was performed. As a result, the first three important features were selected to build the model (Figure 3).

### Predictive model building and model validation

Five classification algorithms were used, namely, RF, eXtreme Gradient Boosting (XGBoost), logistic regression (LR), support vector machines (SVMs), and k-nearest neighbors (KNNs). The hyperparameters for each classifier were tuned *via* a grid search process to maximize the model's performance. The two groups of patients were randomly assigned; 70% to the training set (*N* = 165) and 30% to the testing set (*N* = 66). Due to the limited sample size of the dataset, the 5-fold cross-validation method was used to train the training set and the best threshold corresponding to the maximum Youden's index was selected in the training set and applied in the testing set (33, 34). The models were validated in the testing set (33, 34). The 95% confidence intervals of the training and testing sets were obtained using bootstrap methods (repeated 1,000 times) (35). For each of these five different algorithms, the screened radiomics, clinical, and combined features information mentioned above were used to construct models, respectively.

### Statistical analysis

Continuous variables were recorded as mean ± standard deviation, while categorical variables were recorded as percentages. The results were compared using a *t*-test for continuous variables and a χ^2^ test for categorical variables. The percentages of missing values were as followed: fibrinogen (18.7%) and homocysteine (21.9 %). Missing values were filled with the mean values.

The model prediction performance was determined based on the accuracy, precision, specificity, and sensitivity test. The receiver operating characteristic curve (ROC) was constructed, and the area under the ROC curve (AUC) was presented as the model's predictive ability. The Delong test checked the different feature sets in similar models (6). All statistical analysis procedures were performed using Python (version 3.7.6). The *P* < 0.05 was considered to be statistically significant.

## Results

### Clinical characteristics

**Figure 4:**
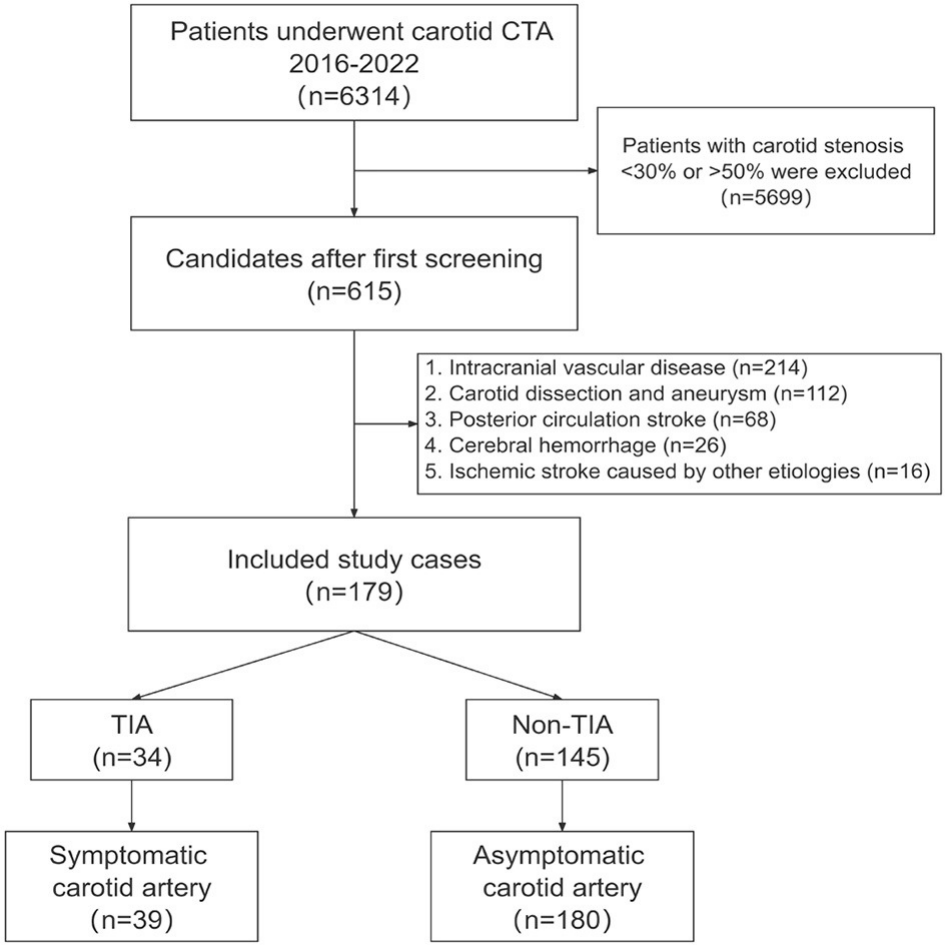
Patient selection. The flowchart shows the process of enrolling patients in this study. The original population consisted of 6,314 subjects who had undergone carotid CTA studies, and we first screened the 615 subjects with mild carotid stenosis. According to the inclusion and exclusion criteria, we finally enrolled 179 subjects and included patients with TIA after CTA in the symptomatic group. CTA, computed tomography angiography; TIA, Transient ischemic attack.

Baseline characteristics and imaging CTA of 615 patients were initially obtained. Under the specified exclusion conditions, 112 who had carotid dissection and aneurysm, 214 who had intracranial vascular disease, 68 who had posterior circulation stroke, 26 who had a cerebral hemorrhage, and 16 who had an ischemic stroke caused by other etiologies were all excluded. The final study included 179 patients (mean age, 65.4 years; 69.9% males), and a total of 219 carotidarteries were included in the final analysis, of which 39 had a TIA (Figure 4).

### Analysis of clinical features, radiomics features, and combined features

After we extract and select the feature information, the first nine features selected for the radiomics mode were maximum 3D diameter, major axis length, maximum 2D diameter (column), maximum 2D diameter (row), inverse variance, maximal correlation coefficient, gray level variance, strength, and voxel volume. The first two features selected for the clinical model were LDL and UA. The first three features selected for the combined model were maximum 3D diameter, LDL, and UA.

### Predictive model evaluation

**Figure 5:**
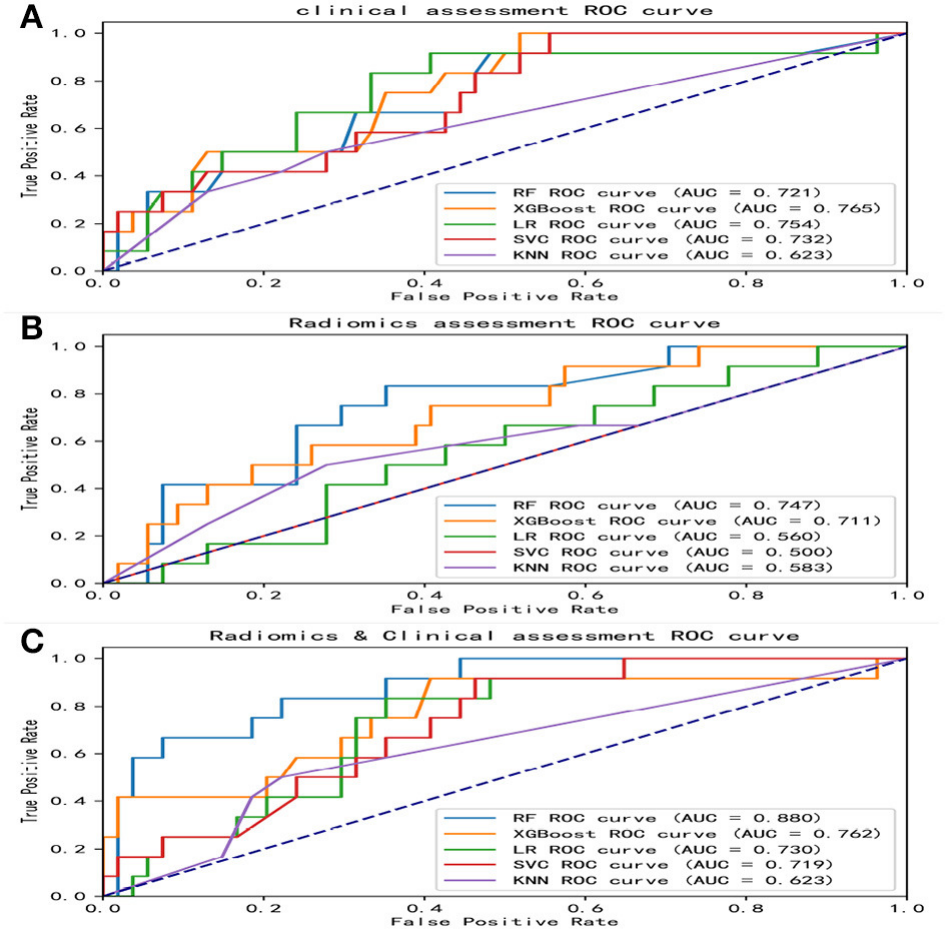
ROC curves of various models. The area under the ROC curves (AUCs) of five different machine learning models constructed from radiomics features, clinical features, and combined features, respectively. **(A)** ROC curves of five machine learning models built with clinical features. **(B)** ROC curves of five machine learning models built with radiomics features. **(C)** ROC curves of five machine learning models built with combined features. ROC, receiver operating characteristic; AUC, area under curve; RF, random forest; XGBoost, eXtreme gradient boosting; LR, logistic regression; SVM, support vector machines; KNN, k-nearest neighbors.

**Figure 6:**
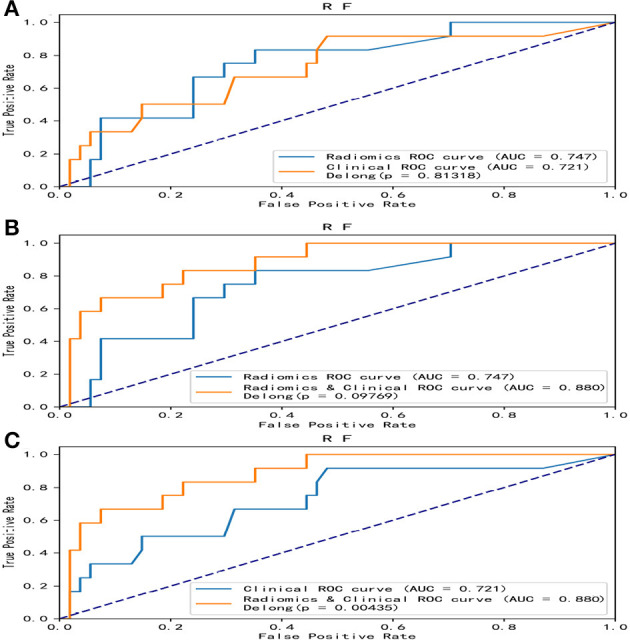
Comparative analysis of ROC in the random forest model. Among the five models, the random forest model showed the best predictive performance. The random forest model was constructed with radiomics features, clinical features, and combined features, respectively, and the DeLong test was performed. The combined model was significantly better than the clinical model. **(A)** A DeLong test was used to compare the radiomics model and clinical model. *P* = 0.813. **(B)** A DeLong test was used to compare the radiomics model and the combined model. *P* = 0.098. **(C)** The DeLong test was used to compare the clinical model and the combined model. *P* = 0.004. ROC, receiver operating characteristic; RF, random forest.

**Figure 7:**
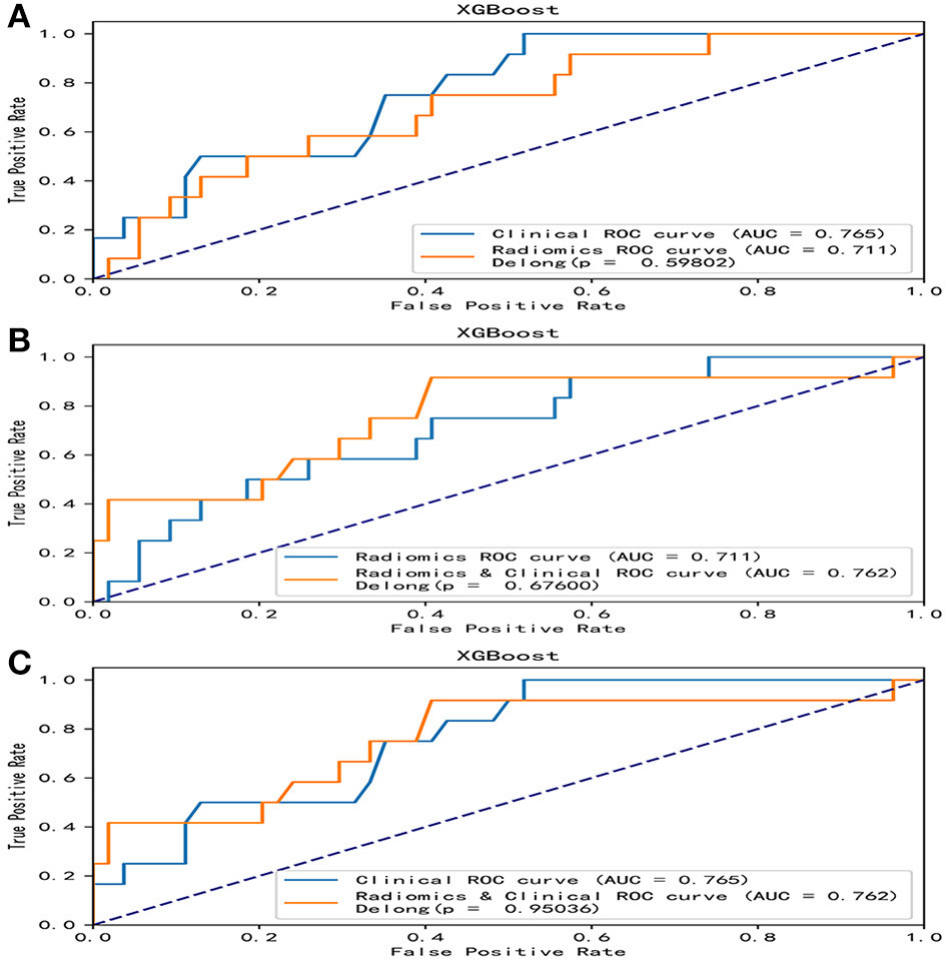
Comparative analysis of ROC in the XGBoost model. In the XGBoost model, there were no statistical differences in the models based on clinical features, radiomics features, and combined features. **(A)** A DeLong test was used to compare the clinical model and the radiomics model. *P* = 0.598. **(B)** A DeLong test was used to compare the radiomics model and the combined model. *P* = 0.676. **(C)** The DeLong test was used to compare the clinical model and the combined model. *P* = 0.950. ROC, receiver operating characteristic; RF, random forest.

Overall, the RF model showed good predictive ability, followed by the XGBoost model, while the remaining three models had poor predictive performance (Figure 5). The top nine important features of radiomics data were selected, and the models were constructed and evaluated in the training and testing sets, respectively, which showed good diagnostic performance in the training and testing sets. The best model was RF (training set: AUC = 0.982, 95%CI, 0.973–0.999, ACC = 0.973, precision = 0.878, sensitivity = 0.968, and specificity = 0.865; testing set: AUC = 0.746, 95%CI, 0.761–0.979, ACC = 0.787, precision = 0.556, sensitivity = 0.417, and specificity = 0.926), and the performance of other models is shown in Table 2. In the machine learning models based on clinical features, the first two important features were LDL and UA, and the models were constructed and evaluated in the training and testing sets, respectively. The best model was XGBoost (training set: AUC = 0.948, 95%CI, 0.921–0.981, ACC = 0.936, precision = 0.828, sensitivity = 0.762, and specificity = 0.841; testing set: AUC = 0.765, 95%CI, 0.574–0.847, ACC = 0.727, precision = 0.429, sensitivity = 0.500, and specificity = 0.852). In the machine learning model based on combined features, the first three features were maximum 3D diameter, LDL, and UA, respectively, and the best model was RF (training set: AUC = 0.983, 95%CI, 0.977–0.998, ACC = 0.988, precision = 0.882, sensitivity = 0.952, and specificity = 0.873; testing set: AUC = 0.879, 95%CI, 0.787–0.979, ACC = 0.863, precision = 0.778, sensitivity = 0.583, and specificity = 0.963). In conclusion, the RF model constructed based on radiomics features and clinical features achieved the best predictive performance. We used the Delong test (goodness of fit test) to check whether different data were statistically different on the same model. In the RF model, the clinical information-based model was not statistically different from the radiomics-based model (AUC = 0.721 vs. AUC = 0.746, *p* = 0.813). However, the combined model outperformed the model based on clinical information (AUC = 0.879 vs. AUC = 0.721, *p* = 0.004) (Figure 6). In the XGBoost model, the models established by the three data types were not statistically different from each other (Figure 7).

**Table 2:** Performance of predictive models.

**Models**	**Datasets**	**AUC**			**Accuracy**			**Precision**			**Sensitivity**			**Specificity**		
		**Clinic**	**Radiomic**	**Clinic + Radiomic**	**Clinic**	**Radiomic**	**Clinic + Radiomic**	**Clinic**	**Radiomic**	**Clinic + Radiomic**	**Clinic**	**Radiomic**	**Clinic + Radiomic**	**Clinic**	**Radiomic**	**Clinic + Radiomic**
RF	Training set	0.981	0.982	0.983	0.980	0.973	0.988	0.873	0.878	0.882	0.929	0.968	0.952	0.865	0.865	0.873
	Testing set	0.721	0.746	0.879	0.787	0.787	0.863	0.428	0.556	0.778	0.500	0.417	0.583	0.851	0.926	0.963
XGB	Training set	0.948	0.957	0.960	0.936	0.952	0.968	0.828	0.824	0.801	0.762	0.929	0.960	0.841	0.802	0.762
	Testing set	0.765	0.711	0.761	0.727	0.757	0.727	0.429	0.444	0.308	0.500	0.333	0.667	0.852	0.907	0.667
LR	Training set	0.778	0.734	0.796	0.746	0.706	0.698	0.717	0.682	0.977	0.865	0.698	0.992	0.659	0.675	0.976
	Testing set	0.754	0.560	0.730	0.697	0.667	0.667	0.313	0.250	0.323	0.833	0.417	0.833	0.593	0.722	0.611
SVM	Training set	0.989	0.500	0.979	0.710	0.500	0.968	0.944	0.632	0.984	0.944	0.952	0.992	0.944	0.444	0.984
	Testing set	0.732	0.500	0.718	0.515	0.182	0.788	0.444	0.182	0.500	0.333	1.000	0.083	0.907	0.000	0.981
KNN	Training set	0.905	0.797	0.868	0.968	0.746	0.940	0.935	0.632	0.867	0.794	0.952	0.825	0.944	0.444	0.873
	Testing set	0.623	0.583	0.623	0.712	0.682	0.742	0.429	0.182	0.500	0.250	1.000	0.083	0.926	0.000	0.981

AUC, area under curve; RF, random forest; XGB, eXtreme gradient boosting; LR, logistic regression; SVM, support vector machine; KNN, k-nearest neighbors.

## Discussion

In this study, patients with mild carotid stenosis (30%−50% NASCET) had a cumulative incidence of 17.8% of TIA on the ipsilateral side of the carotid artery after follow-up. This is slightly higher than previously reported results (10–15%) (3), possibly because living above 3,500 meters appears to be associated with a significantly increased risk of ischemic stroke and TIA, which may be related to polycythemia and other related factors such as increased blood viscosity (36).

Radiomics refers to the extraction of information from imaging images, including images that are difficult to identify by the human eye. Radiomics can capture tissue and lesion characteristics, thus, radiomics was first used to analyze tumor aggressiveness. One study predicted axillary lymph node metastasis in breast cancer. The XGBoost algorithm was used to establish a prediction model, and its AUC was 0.890 (20). Radiomics can also be used to predict clinical endpoints such as survival and treatment response. Nazari et al. predicted the mortality risk of patients with clear cell renal cell carcinoma within 5 years. They combined radiomics features with clinical data to establish a predictive model using four machine learning algorithms, of which the XGBoost model showed the best performance (AUC was in the range of 0.95~0.98) (37). Radiomics signatures can be mined, and in sufficiently large data, radiomics can be used to discover previously unknown markers and models of disease development, progression, and response to treatment, as well as new imaging studies for known diseases' classification. Liu et al. classified patients with rectal cancer and predicted the clinical outcomes of patients (38). Recent studies on radiomics and machine learning primarily focus on tumor classification or the prediction of clinical endpoints. According to the literature, there is no relevant research on the use of machine learning to build a model to predict the complications of patients with carotid artery stenosis of 30–50% based on CT imaging and clinical laboratory results; hence, the motivation for this study apply CT radiomics features combined with clinical features to predict the risk of TIA in patients with mild carotid stenosis.

In this study, we trained RF, XGBoost, LR, SVM, and KNN five radiomics group models on carotid artery CTA images to predict the occurrence of cerebrovascular symptoms in participants. When we evaluated our results in the testing set, an RF model based on radiomics and clinical information identified symptomatic participants with optimal diagnostic accuracy. Furthermore, we demonstrate that, although the radiomics-based RF model was not statistically significantly different when compared with the assessment of clinical characteristics, the combined information-based RF model could well distinguish between the high-risk and low-risk groups from participating (AUC = 0.879 vs. AUC = 0.721, *p* = 0.004).

In addition to predicting TIA in carotid artery stenosis, this study can also provide a valuable reference for formulating treatment plans through prediction. The ESVS issued a set of guidelines proposing medical therapy as an intervention for asymptomatic patients with stenosis <60% and symptomatic patients with stenosis <50%, and subsequently indicated that if symptoms persist, despite the absence of BMT, it may be reasonable to consider CEA/CAS (3). Therefore, there remains uncertainty in the treatment plan of patients with stenosis of 30–49%. If the patient predicts the risk of TIA in the future through the model, under the premise of BMT, CEA/CAS surgery can be given a higher priority to avoid complications. Therefore, we performed radiomics feature analysis of carotid arteries and their plaques, which have the potential ability to identify plaques leading to the risk of cerebrovascular events and have instructive value in guiding and optimizing the management of patients with mild stenosis. We aim to establish a model with optimal performance in predicting complications, combining both radiomics and clinical features. The models built together have the best predictive performance and are statistically different from the clinical features model. Traditional CTA imaging can only provide limited information on carotid plaque characteristics, and we need more objective methods that rely less on medical expertise while increasing prediction accuracy. Radiomics has the potential to be a tool that facilitates accurate phenotyping of abnormalities based on radiological images (39). A previous study explored that CT texture features could distinguish between symptomatic and asymptomatic patients, with AUCs ranging from 0.68 to 0.81 (40). The study used radiomics to acquire extensive data on stenotic carotid arteries and utilized machine learning (ML) models to improve diagnostic performance. The results showed that ML is beneficial for carotid CTA plaque analysis. However, the assessment of the carotid artery was not performed using clinical information added to the model. Another study on CTA used a machine learning model to identify symptomatic participants using radiology and routine assessment. Routine assessment refers to the analysis of carotid artery stenosis, plaque length, plaque thickness, and plaque ulceration, combined with imaging features, to identify symptomatic participants. The AUC was 0.85 (39). ML models based on radiomics and clinical features can be an ideal tool to identify which specific CTA plaque features are associated with increased TIA risk. Our study uses radiomics features and clinical laboratory tests (triglycerides, low-density lipoprotein, homocysteine, etc.) to assess the risk of complications based on machine learning models.

Although the findings are promising, some limitations must be addressed. The cases in this study were collected from a single-center. We included a small number of cases, and the incidence of cerebral ischemic events in patients with mild carotid stenosis was relatively low. The number of negative patients was much larger than that of positive patients, which may bias our results. To overcome overfitting our model, we calculated all diagnostic scores using 5-fold cross-validation. Prospective studies with larger sample sizes are still needed to assess long-term accuracy and stability. Manual segmentation may also affect parameter values, while automatic segmentation may reduce inter-observer variability and improve the feasibility of applying these methods on larger datasets.

## Conclusion

In conclusion, we established five models for predicting ischemic cerebrovascular events in patients with mild carotid stenosis using radiomics, clinical data, and combined data based on machine learning models. After comparison, the random forest model of the combined data showed the best accuracy and maybe a more comprehensive and specific prediction model than previously reported methods. According to the predicted results, patients can have improved guided treatment plans using the proposed model.

## Data availability statement

The raw data supporting the conclusions of this article will be made available by the authors, without undue reservation.

## Ethics statement

The studies involving human participants were reviewed and approved by the Ethics Committee of The First Affiliated Hospital of Kunming Medical University (No. 2022-274). Written informed consent from the patients/participants or patients/participants legal guardian/next of kin was not required to participate in this study in accordance with the national legislation and the institutional requirements.

## Author contributions

HX and LY: conception and design. YF: administrative support. WZ and JH: provision of study materials or patients. HX, YL, and YB: collection and assembly of data. HX, CZ, LY, and LZ: data analysis and interpretation. All authors: manuscript writing and final approval of manuscript.
